# Mimicking lichens: incorporation of yeast strains together with sucrose-secreting cyanobacteria improves survival, growth, ROS removal, and lipid production in a stable mutualistic co-culture production platform

**DOI:** 10.1186/s13068-017-0736-x

**Published:** 2017-03-21

**Authors:** Tingting Li, Chien-Ting Li, Kirk Butler, Stephanie G. Hays, Michael T. Guarnieri, George A. Oyler, Michael J. Betenbaugh

**Affiliations:** 1 Department of Chemical and Biomolecular Engineering, Johns Hopkins University, Baltimore, MD 21218 USA; 2 Department of Biophysics, Johns Hopkins University, Baltimore, MD 21218 USA; 3 Department of Systems Biology, Harvard Medical School, Boston, MA 02115 USA; 4 Wyss Institute for Biologically Inspired Engineering, Harvard University, Boston, MA 02115 USA; 5 National Bioenergy Center, National Renewable Energy Laboratory, Golden, CO 80401 USA

**Keywords:** Cyanobacteria, Yeasts, Co-culture, Sucrose, ROS, Artificial lichen, Hydrogen peroxide, Lipid production

## Abstract

**Background:**

The feasibility of heterotrophic–phototrophic symbioses was tested via pairing of yeast strains *Cryptococcus curvatus*, *Rhodotorula glutinis*, or *Saccharomyces cerevisiae* with a sucrose-secreting cyanobacterium *Synechococcus elongatus*.

**Results:**

The phototroph *S. elongatus* showed no growth in standard BG-11 medium with yeast extract, but grew well in BG-11 medium alone or supplemented with yeast nitrogen base without amino acids (YNB w/o aa). Among three yeast species, *C. curvatus* and *R. glutinis* adapted well to the BG-11 medium supplemented with YNB w/o aa, sucrose, and various concentrations of NaCl needed to maintain sucrose secretion from *S. elongatus*, while growth of *S. cerevisiae* was highly dependent on sucrose levels. *R. glutinis* and *C. curvatus* grew efficiently and utilized sucrose produced by the partner in co-culture. Co-cultures of *S. elongatus* and *R. glutinis* were sustained over 1 month in both batch and in semi-continuous culture, with the final biomass and overall lipid yields in the batch co-culture 40 to 60% higher compared to batch mono-cultures of *S. elongatus.* The co-cultures showed enhanced levels of palmitoleic and linoleic acids. Furthermore, cyanobacterial growth in co-culture with *R. glutinis* was significantly superior to axenic growth, as *S. elongatus* was unable to grow in the absence of the yeast partner when cultivated at lower densities in liquid medium. Accumulated reactive oxygen species was observed to severely inhibit axenic growth of cyanobacteria, which was efficiently alleviated through catalase supply and even more effectively with co-cultures of *R. glutinis*.

**Conclusions:**

The pairing of a cyanobacterium and eukaryotic heterotroph in the artificial lichen of this study demonstrates the importance of mutual interactions between phototrophs and heterotrophs, e.g., phototrophs provide a carbon source to heterotrophs, and heterotrophs assist phototrophic growth and survival by removing/eliminating oxidative stress. Our results establish a potential stable production platform that combines the metabolic capability of photoautotrophs to capture inorganic carbon with the channeling of the resulting organic carbon directly to a robust heterotroph partner for producing biofuel and other chemical precursors.

**Electronic supplementary material:**

The online version of this article (doi:10.1186/s13068-017-0736-x) contains supplementary material, which is available to authorized users.

## Background

Most microbes live in complex interacting heterogeneous populations in nature, including the gut microbiota of insects and animals, waste sludge, and lichens. Lichens, composed of photoautotrophs and heterotrophs, provide an extremely stable and self-supporting symbiosis [1, 2]. These autotroph–heterotroph symbionts thrive by performing a wide variety of functions that combine the attributes and metabolic pathways of multiple microbes. Carbon needed for biosynthesis is typically provided by the photobiont (cyanobacteria or green algae) and supplied to the heterotroph. In turn, the heterotrophic symbionts, typically fungi, provide minerals and nutrients, as a result of the vast array of metabolites in their secondary metabolism, as well as carbon dioxide, water, and protection from the environment [3–5]. Furthermore, these relationships offer a division of labor to each participant by allowing the photobiont to perform carbon dioxide (CO_2_) and energy capture and the heterotroph to accumulate biomass and produce complex metabolites. This differentiation allows the community to function most efficiently by allowing each organism to perform specific tasks. However, the cultivation of natural lichens remains challenging and the exact nature of these complex interactions among lichens is not well known or characterized.

Phototrophs and heterotrophs exhibit mutualistic, competitive, and allelopathic interactions. Heterotrophic bacteria related to *Pseudomonas* and isolated from cyanobacterial mat led to an eightfold increase in the cyanobacterial biomass accumulation when co-cultured together with the cyanobacterium *Synechocystis* PCC6803 [6]. A marine cyanobacterium *Prochlorococcus* has been pair-wise co-cultured with hundreds of marine heterotrophic bacteria, some of which enabled *Prochlorococcus* to grow faster and reach higher final culture chlorophyll fluorescence. In contrast, some strains showed antagonistic interactions with *Prochlorococcus* and several displayed strong growth inhibition on *Prochlorococcus* [7]. Differential interactions have also been reported in green microalgal consortia [8–10]. Co-culture of the green algae *Chlorella* with isolates from a microbial consortium resulted in 0.5-3 times greater algal growth than that of algal cells alone, and the growth of the microbial isolates was also promoted in co-culture with algae [8]. On the other hand, the interactions between *Chlorella* and heterotrophic microbes can exhibit variable patterns, in which some co-cultured bacteria inhibited algal growth [10], and some strains had no influence [9]. Therefore, the physiological behaviors and interactions between phototrophs and heterotrophic partners in microbial consortia can vary and the exact interaction is not always evident.

Specific synergistic relationships between photoautotrophs and heterotrophs have been observed. Exchange of CO_2_ and O_2_ gas between phototrophs and their heterotrophic partners often occurs. For example, dissolved oxygen in a pure culture of yeast increases dramatically when microalgae is supplied [11, 12], and the oxygen generated by microalgae benefits the co-cultured heterotrophic microorganisms for biomass and lipid production [13, 14]. Alternatively, oxygen removal by a heterotrophic partner can facilitate photosynthesis and lipid production in microalgae. High oxygen accumulation imposes an impediment on algal growth by inhibiting photosynthesis, which can be especially problematic in closed photobioreactors [15]. Minimizing dissolved oxygen in the culture medium has been shown to enhance the lipid production of the green alga *Chlorococcum littorale* under photoautotrophic conditions [16]. In turn, the heterotrophic partner supplies the microalgae with additional CO_2_ derived from several metabolic processes.

The members present in phototrophic–heterotrophic symbiosis, such as lichens, sometimes include cyanobacteria, which can convert CO_2_ and sunlight energy into useful metabolites. These cyanobacteria can produce soluble organic carbon, including glucose and sucrose as a result of photosynthesis [17–19]. Sucrose accumulation in some cyanobacteria occurs as a cellular response to salt stress. Previously, the cyanobacterium *Synechococcus elongatus* PCC7942 has been engineered to effectively secrete intracellular sucrose in the culture medium by over-expressing the *Escherichia coli* gene *cscB*, which encodes a proton and sucrose symporter [20]. Addition of 200 mM NaCl in the culture medium induced about 80% of biomass generated as extracellular sucrose. Furthermore, both cell expansion and sucrose production occurs simultaneously, and the ratio of cellular biomass to sucrose is adjustable via changing the osmotic pressure in the culture medium [20]. This characteristic makes sucrose-secreting *S. elongatus* a promising candidate for providing carbon to a co-cultured heterotrophic partner in a mutualistic co-culture pairing as shown in previous experiments [21, 22].

One potential benefit of photobiont–heterotroph co-culture is the production of potential biofuel precursors. Oleaginous yeasts such as *Cryptococcus curvatus* and *Rhodotorula glutinis*, with their ability to channel various carbon feeds into complex secondary metabolites, have proven to be generators of biofuel precursors [23–25], with *C. curvatus* and *R. glutinis* accumulating up to 69 and 72% lipid, respectively [26, 27]. However, in order to synthesize advanced biofuels products, oleaginous yeasts must be fed with organic carbon feedstocks, which adds processing steps and increases costs. The wide abundance of cellulosic materials represents one alternative feedstock, but these often require additional pre-treatment steps [28, 29] and can lead to toxic culture environments [30, 31].

Co-culture fermentation of cyanobacteria generating sugar as substrate represents a potentially viable alternative to cellulosic feedstocks. Such an approach provides a division of labor and symbiotic interactions between the photobiont and heterotrophs, in which the chemical precursors for biofuel production could be generated. Therefore, the goal of this study was to establish a synthetic system mimicking lichens by pairing phototrophic sucrose-secreting *S. elongatus* together with yeast strains capable of producing metabolic byproducts, including fatty acid biofuel precursors. Equally important was an examination to determine if the presence of the yeast heterotrophic partner could be beneficial to and symbiotic with the phototrophic partner. In this way, we can begin to establish stable phototrophic–heterotrophic partnerships which represent a potentially sustainable production platform for biofuels and other products by combining the light and CO_2_ harvesting capability of a phototroph with the robust metabolic performance and varied capabilities of heterotrophic yeast.

## Methods

### Growth conditions and assays

Inoculum culture of sucrose-secreting cyanobacterium, cscB^+^ *S. elongatus* PCC 7942 [20], was prepared in 250 mL Erlenmeyer flasks containing 100 mL BG-11 medium supplemented with 1 g/L HEPES (pH 8.9) and 100 mM NaCl. Flasks were incubated at 28 °C agitated with filter-sterilized air that was enriched with CO_2_ to 1% (v/v). Illumination was provided by cool-white fluorescent lamps to give a light intensity of 65 μmol/m^2^ s with 16:8 h light/dark cycle. For experimental cultures (pure cultures of *S. elongatus* and co-cultures of *S. elongatus* together with yeasts), exponentially growing inoculum was transferred into each flask containing BG-11 medium (1.6 × 10^7^/mL in initial culture, if no specific statement), which was supplemented with 3 g/L HEPES (pH 8.9), 1.2 g/L yeast nitrogen base without amino acids (YNB w/o aa), 4 mM ammonium chloride, 1 mM IPTG, and 100 mM NaCl (denoted BG-11[co]). Flasks were weighed at the setup of the time course experiments, and water was added prior to each sampling to correct for evaporation. Cultures were maintained under illumination of 100 μmol/m s with 16:8 h light/dark cycle.

Yeast strains *C. curvatus* (ATCC 20509), *R. glutinis* (ATCC 204091), and *Saccharomyces cerevisiae* (ATCC 204508) were grown overnight in the YPD medium containing 10 g/L yeast extract (YE), 20 g/L peptone, and 20 g/L glucose. YPD cultures were then inoculated (2%, v/v) into 20 g/L sucrose supplemented BG-11[co] medium for seed preparation, maintaining within log phase growth under 65 µmol m^2^ s^‒1^ constant light at 28 °C and 200 rpm in 150 mL flasks. The yeast seeds were washed twice with BG-11 medium before inoculation, and the initial inocula were 0.01 OD_600_ for *C. curvatus* and *R. glutinis*, and 0.03 OD_600_ for *S. cerevisiae*.

### Quantification of cyanobacteria and yeasts

For axenic culture of yeasts, optical density was measured spectrophometrically at 600 nm (OD_600_). In co-culture, serial dilutions were plated on YPD plates to determine yeast viability. Colony-forming units (CFU) were counted after 2 days’ incubation at 25 °C.

*Synechococcus elongatus* concentration was analyzed with a FACSCalibur flow cytometer (BD Biosciences, San Jose, CA) after adding with BD Liquid Counting Beads (BD Biosciences, San Jose, CA). The absolute cell numbers in samples were determined by comparing cellular events to the beads events measured by the flow cytometer using the equation provided in the kit’s TDS document. *S. elongatus* viability was determined by staining with dye solutions and adding with fluorescent counting beads in Cell Viability Kit (BD Biosciences, San Jose, CA) prior to flow cytometry, following the manufacturer’s instructions.

### Measurements and analysis

After pelleting cells, sucrose was determined directly from the supernatant fraction with a Biochemistry Analyzer YSI 2700 Select (YSI Incorporated, Yellow Springs Instrument Co., Inc., Ohio) equipped with a sucrose (YSI 2703) membrane and standardized with 5.0 g/L standard sucrose (YSI 2780) solutions. The pH of the samples was determined using a pH meter (Accumet Model 15, Fisher Scientific) using a combination pH electrode with silver/silver chloride reference (Fisher Scientific), which had been previously standardized to pH 4, 7, and 10. Hydrogen peroxide concentration in the culture was quantified with the Fluorimetric Hydrogen Peroxide Assay Kit (MAK165, Sigma-Aldrich, St. Louis, MO, US) according to manufacturer’s instruction. To determine biomass dry weight (DW), cell suspension sample was centrifuged at 4000 rpm for 5 min, washed twice with distilled water, and then lyophilized by freeze dry vacuum (LGJ-25, Xiangyi, China). Total lipids and lipid profile were analyzed with gas chromatography, using heptadecanoic acid (C17:0) as an internal standard. The corresponding chromatographic conditions and the extraction/transesterification method are described in Dong et al. [32].

### Statistics

Three biological replicates were performed for all data collection, and the statistical tests for significance were determined via a two-tailed *t* test, with a significance level of 0.05, unless stated otherwise.

## Results and discussion

### Axenic growth of different yeast strains

Previously, sucrose permease, *cscB,* expressing *S. elongatus* cells were engineered to efficiently secrete sucrose into the culture medium. The sucrose production is dependent upon osmotic pressure, alkaline environment, and supply of IPTG inducer [20]. To examine if various yeasts could grow well with sucrose as the carbon source, and under the same media conditions in which *S. elongatus* cells grow and produce sucrose, yeast strains *R. glutinis*, *C. curvatus*, and *S. cerevisiae* were cultured individually in modified BG-11 medium.

**Fig. 1:**
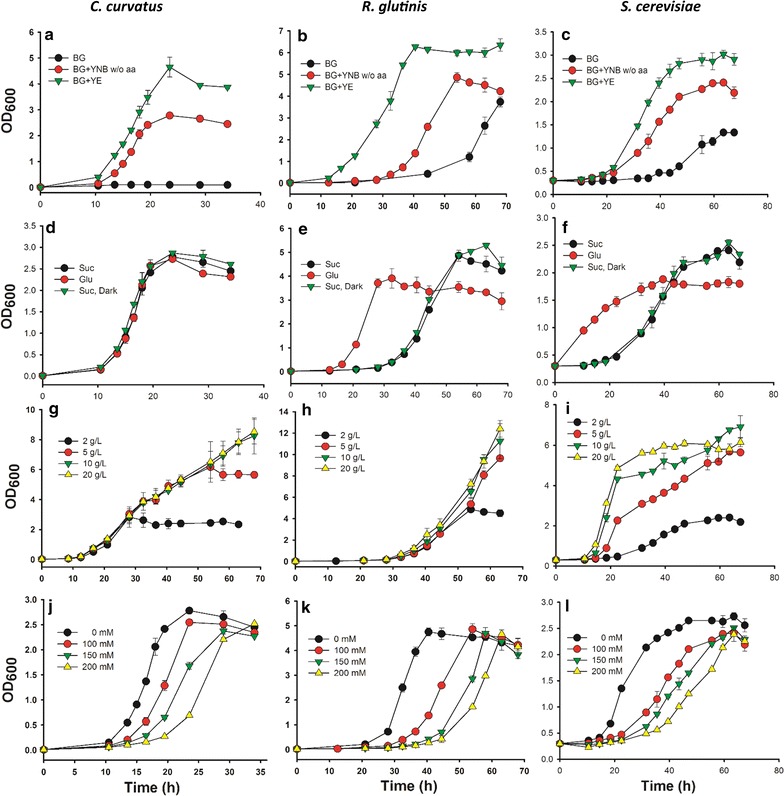
Axenic growth of different yeast strains. **a**–**c** Effect of medium components on monoculture of yeasts; “BG” indicates BG-11 added with 2 g/L sucrose, 4 mM ammonium chloride, 1 mM IPTG, and 100 mM NaCl; “BG + YNB w/o aa” indicates “BG” supplied with YNB w/o aa; “BG + YE” indicates “BG” supplied with YE. **d**–**f** Effect of glucose/sucrose and light/dark on monoculture of yeasts; Medium used here was BG-11[co] supplied with 2 g/L sucrose or 2 g/L glucose, as indicated in the legends. **g**–**i** Effect of sucrose concentration on monoculture of yeasts; Medium used here was BG-11[co] supplied with various concentration of sucrose, as indicated in the legends. **j**–**l** Effect of NaCl concentration on monoculture of yeasts. Medium here was BG-11[co] supplied with 2 g/L sucrose, but with adjusted NaCl concentration in each condition. **a**, **d**, **g**, **j** for *C. curvatus*; **b**, **e**, **h**, **k** for *R. glutinis*; **c**, **f**, **i**, **l** for *S. cerevisiae*. Light condition was used if no specific statement. All data are averages of biological triplicates ± standard deviation

The yeast *C. curvatus* could not grow in BG-11[co] medium supplemented with sucrose only (Fig. 1a). Alternatively, *R. glutinis* and *S. cerevisiae* cells grew in these conditions only after a long lag phase (Fig. 1b, c). However, all the three yeast strains performed well in 2 g/L of sucrose complemented BG-11[co] medium containing either yeast extract (YE) or yeast nitrogen base without amino acids (YNB w/o aa). YE yielded the best growth of each strain, with the highest cell density over the duration of the culture.

Typically, wild-type yeast cells require YE for growth, which provides nitrogenous compounds, carbon, sulfur, trace nutrients, vitamins, and other important growth factors. However, the proposed partner strain *S. elongatus* showed no growth in BG-11 medium with added YE (see Additional file 1), but could grow well in BG-11 medium supplemented with or without YNB w/o aa. The growth of a diverse array of cyanobacteria is inhibited by exogenous metabolites such as amino acids [33–35], although endogenous synthesis is obligatory. For example, glutamine, lysine, as well as histidine inhibited the growth of *Synechocystis* PCC6803 [35]. Therefore, YNB w/o aa instead of YE was adopted as component supplement in BG-11[co] medium for yeast growth.

The above results in Fig. 1a–c indicated that the three wild-type yeast strains can consume sucrose as a sole carbon source, and no sucrose remained in the final culture medium (data not shown). As glucose is the most ubiquitous carbon source for yeast in laboratories, growth on sucrose was compared to this carbon source. *C. curvatus* exhibited similar growth with sucrose as a substrate compared with glucose; while *R. glutinis* and *S. cerevisiae* exhibited a prolonged lag phase with sucrose (Fig. 1d–f). Additionally, illumination had little influence on the growth of the three yeast strains, which indicates compatibility of yeast strains with phototrophs under light condition.

To further evaluate if yeast cells could grow on a sucrose substrate with a phototrophic partner in a co-culture system, the effect of varying sucrose concentrations was investigated using pure cultures of the three yeast strains in BG-11[co] medium. Growth of *S. cerevisiae* demonstrated dependence on sucrose concentration in the medium as previously reported [21, 22] (Fig. 1i). However, both *C. curvatus* and *R. glutinis* showed similar growth profiles regardless of the sucrose concentrations, although *R. glutinis* exhibited an overall longer lag phase (Fig. 1g, h). A similar delay in the growth phase was observed for the *R. glutinis* when comparing growth on sucrose to growth on glucose (Fig. 1e).

The sucrose production by *cscB* ^+^ *S. elongatus* cells can be tuned via the osmotic pressure imposed on the culture system, with up to ~80% of total mass generated as sucrose at higher salinities [20]. Therefore, we investigated yeast growth with increasing NaCl concentrations (0, 100, 150 and 200 mM) in BG-11[co] medium (Fig. 1j–l). The three yeast strains exhibited similar growth patterns in which the lag phases were extended and the growth rates delayed with increasing salt concentrations (see Additional file 2). Lag phases appeared approximately two times longer for growth of the three yeast strains with 200 mM NaCl compared with no supplemental NaCl supply. To ensure robust growth of the three yeast strains, 100 mM NaCl was used with BG-11[co] medium in co-culture with *S. elongatus* in subsequent studies.

### Axenic growth of *S. elongatus*

**Fig. 2:**
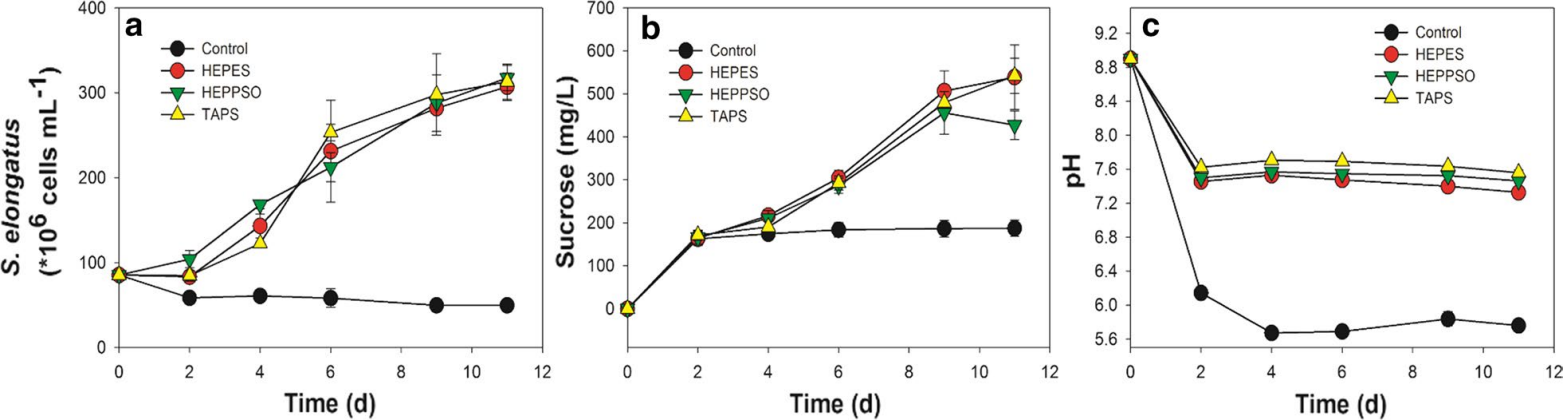
Axenic growth of *S. elongatus* with different pH buffer in the medium. **a** Cell numbers (per mL) of *S. elongatus*; **b** Sucrose (mg/L) secreted by *S. elongatus*; **c** pH value. 3 g/L of different buffers were used in the medium. All data are averages of biological triplicates ± standard deviation

Sucrose export by *cscB* ^+^ *S. elongatus* is dependent upon medium pH, with maximal sucrose export in an alkaline environment [20]. To ensure that sucrose was continuously and efficiently secreted, three different Good’s buffers, HEPES, HEPPSO, and TAPS, were tested for their capability in buffering the axenic growth of cyanobacteria *S. elongatus* cells. As shown in Fig. 2, *S. elongatus* cells did not grow and gradually died in BG-11[co] medium without any buffering agent, in which the pH dropped from an initial value of 8.8 to below 6 after 4 days. For the pH buffered groups, however, the *S. elongatus* cells exhibited similar growth curves and exported the sucrose at comparable rates, with the sucrose level in the medium gradually increasing up until 9 days. Additionally, the three buffers displayed similar pH buffering capabilities and could all maintain pH values above 7.2 over the culture duration. Based on these findings, HEPES buffer was chosen as the buffering agent to be used in following co-culture experiments.

### Co-culture of *S. elongatus* with different yeast strains

**Fig. 3:**
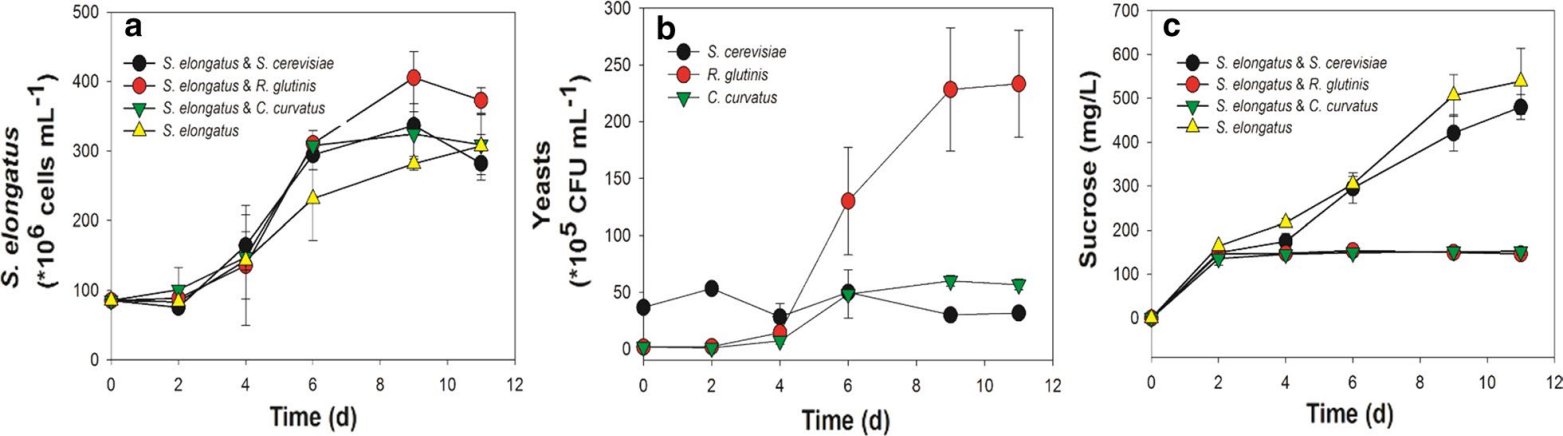
Co-culture of *S. elongatus* with different yeast strains. **a** Cell numbers of *S. elongatus*; **b** CFU numbers of yeasts; **c** Sucrose secreted by *S. elongatus*. All data are averages of biological triplicates ± standard deviation

Following the initial mono-culture experiments, the CscB-expressing *S. elongatus* was next grown in co-culture medium BG-11[co] with the different yeast strains. Shown in Fig. 3 are the cell counts for *S. elongatus* and the three yeast strains, along with sucrose levels. The *S. elongatus* cells grown in co-culture with yeast cells exhibited slightly higher cell densities, on average, than cells in mono-culture in the middle to late exponential phase (6 and 9 days). Among yeast strains, wild-type *S. cerevisiae* displayed no growth in the co-culture consistent with previous findings [22], and actually decreased in cell numbers over the time period, even though the initial CFU numbers inoculated were 20 times larger compared with the other two yeast species. *C. curvatus* showed a modest enhancement in cell numbers from 1.8 × 10^5^ to 5.7 × 10^6^ CFU over the 11 day culture duration. Finally, *R. glutinis* cells grew efficiently in co-culture, reaching cell densities five times higher than the other two cultures. Surprisingly, *R. glutinis* and *C. curvatus* cells utilized similar amounts of sucrose with sucrose levels kept below 200 mg/L in the medium compared to levels above 500 mg/L in the mono-culture of *S. elongatus*. Sucrose levels in the co-culture medium of *S. cerevisiae* with *S. elongatus* showed an accumulation pattern more similar to the mono-culture of *S. elongatus*, with final sucrose levels around 500 mg/L after 11 days, indicating the yeast *S. cerevisae* used in this study could not utilize sucrose at these levels, and likely require higher levels of sucrose for expansion as indicated in Fig. 1 and as demonstrated in the literature [21, 22].

**Fig. 4:**
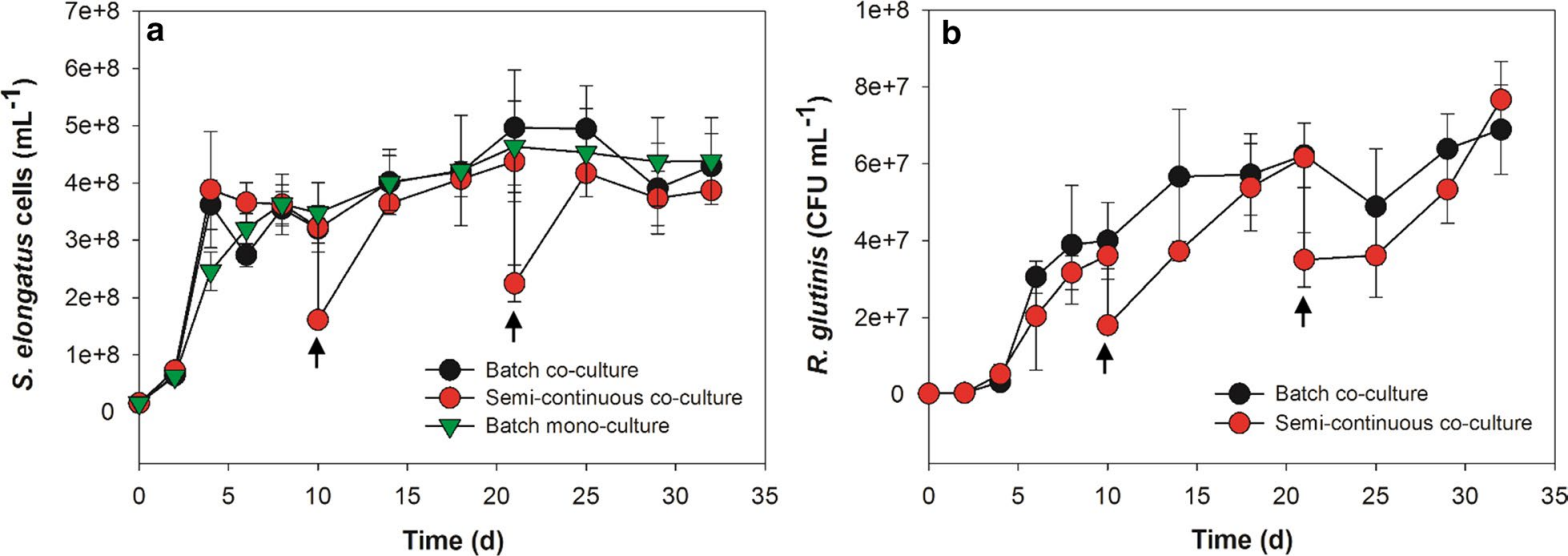
Long-term batch and semi-continuous co-culture of *S. elongatus* and *R. glutinis*. **a** Cell numbers of *S. elongatus*; **b** CFU numbers of yeast *R. glutinis*. *Arrows* indicate the time when 50 mL of original culture was withdrawn and 50 mL of fresh BG-11[co] medium was added. All data are averages of biological triplicates ± standard deviation

In order to evaluate sustainability of a co-culture system composed of cyanobacteria and yeast, long-term batch and semi-continuous cultures of the cyanobacteria *S. elongatus* together with the yeast *R. glutinis* were carried out. After 4 days in batch, the growth of *S. elongatus* plateaued but remained stable for more than 1 month with a marginal increase in densities (Fig. 4a). Meanwhile, the CFU of the yeast *R. glutinis* increased rapidly for 8 days and then increased more gradually in the batch cultures (Fig. 4b). In semi-continuous culture, cyanobacterial and yeast cell numbers were reduced by half through replacement of the original cultures with fresh BG-11[co] medium at 10 and 21 days, respectively, shown in Fig. 4 as arrows. Nonetheless, cell numbers rapidly recovered and eventually attained similar levels to the batch culture. Phototrophic cyanobacteria and heterotrophic yeasts possess different resource acquisition traits, so less competition is observed between members compared with either co-cultures of phototrophs or co-cultures of heterotrophs. For example, acetate-secreting cyanobacterium *Synechococcus* sp. overtook a culture when growing together with the acetate-consuming algae *Chlamydomonas reinhardtii*, and the cyanobacterial cells needed to be encapsulated in alginate beads to slow their growth [36].

Interestingly, the final biomass DW in the batch co-culture was much higher than the batch mono-culture of *S. elongatus* (Table 1). We also examined lipid content in the co-culture versus monoculture. The total fatty acids (TFA) in the batch co-culture was 54% higher than in mono-culture, in part because of the increased biomass from the yeast partner. In semi-continuous culture, the biomass DW and TFA in the final harvested biomass were 650 and 35 mg/L, respectively. With addition of the 50 mL of culture that was removed both at 10 and 21 days, respectively, the total biomass yield finally in semi-continuous culture was 721 mg/L of medium. The TFA was 39 mg/L, which was about 60% above the levels for *S. elongatus* mono-culture. Also, the cultures could be manipulated by nitrogen or other stresses for even higher levels of lipids as desired.

**Table 1:** Biomass DW and total fatty acids (TFA) in batch mono-culture, batch co-culture, and semi-continuous co-culture

	Mono-culture (mg/L)	Co-culture (mg/L)	Semi-continuous culture			
			Final harvest (mg/L)	10st day withdraw (mg in 50 mL)	21st day withdraw (mg in 50 mL)	Total harvest (mg/L)
DW	570.37 ± 3.49	792.36 ± 0.98	650.00 ± 3.93	28.90 ± 0.52	50.30 ± 0.33	721.00 ± 1.02
TFA	24.61 ± 1.49	37.86 ± 0.77	34.66 ± 3.77	1.64 ± 0.02	2.69 ± 0.07	39.02 ± 2.13

All data are averages of biological triplicates ± standard deviation

**Fig. 5:**
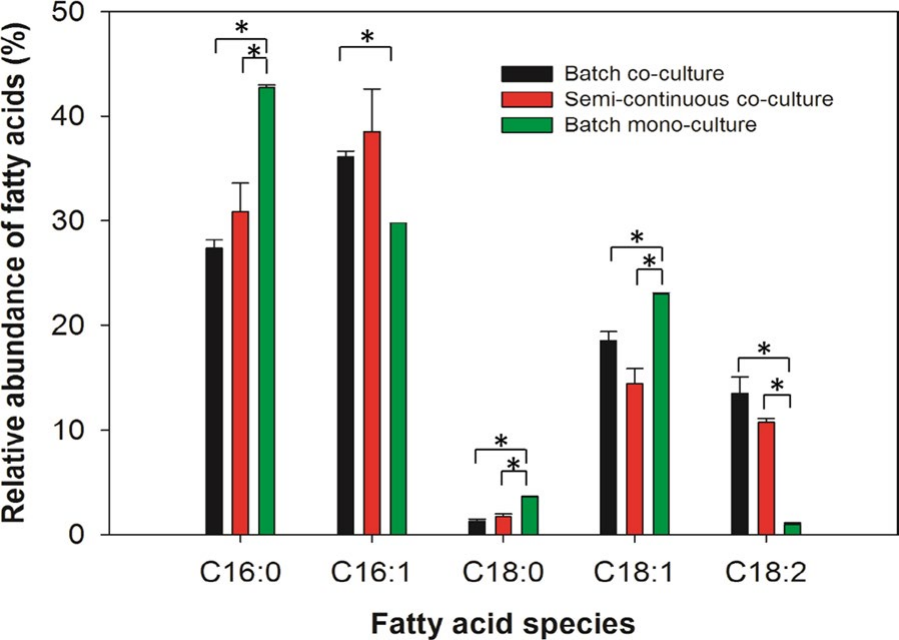
Comparison of fatty acid profiles in different cultures. All data are averages of biological triplicates ± standard deviation. *Significant difference in paired comparisons (*p* < 0.05)

A fatty acid profile analysis revealed that the principal fatty acids produced in the mono- and co-cultures were palmitic (C16:0), palmitoleic (C16:1), stearic (C18:0), oleic (C18:1), and linoleic acid (C18:2) (Fig. 5). While the mono-cultures of *S. elongatus* contained significantly higher levels of palmitic, stearic, and oleic acids, the co-cultures of both batch and semi-continuous culture showed enhanced levels of palmitoleic and linoleic acids. These oils could serve as potential oil feedstocks for biodiesel production that require long-chain fatty acids with 16 and 18 carbon atoms [37].

### Chemical interaction involved in cultures of cyanobacteria with/without yeast

While the yeast *R. glutinis* is clearly dependent on the cyanobacterium for a supply of sucrose for growth, we wanted to explore any impact the yeast may have on the growth and maintenance of *S. elongatus* in HEPES buffered BG-11[co] medium.

**Fig. 6:**
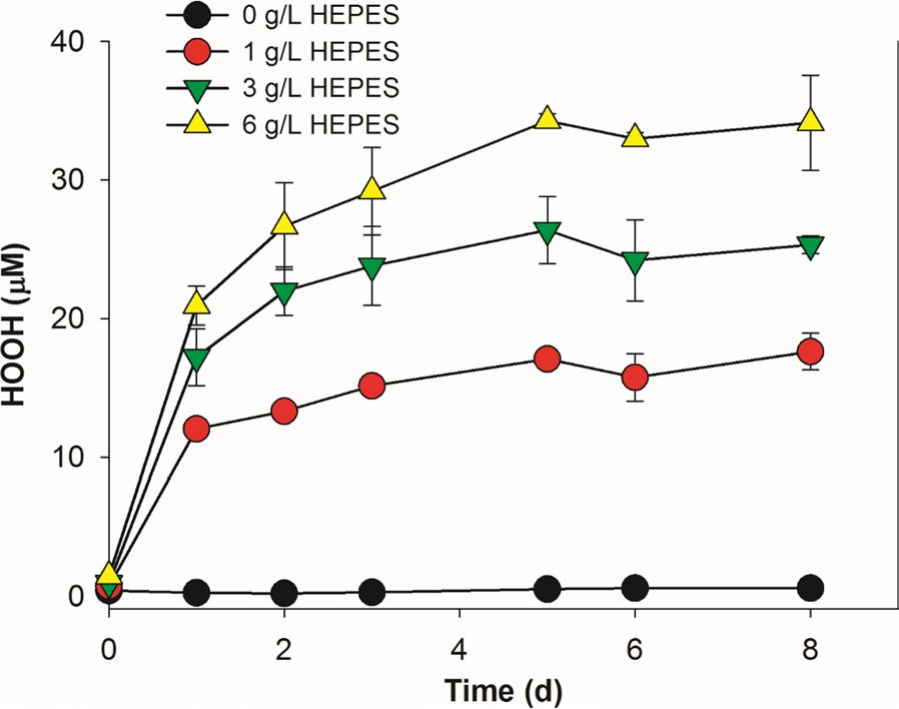
HOOH formation in illuminated BG-11[co] medium containing various concentration of HEPES. All data are averages of biological triplicates ± standard deviation

**Fig. 7:**
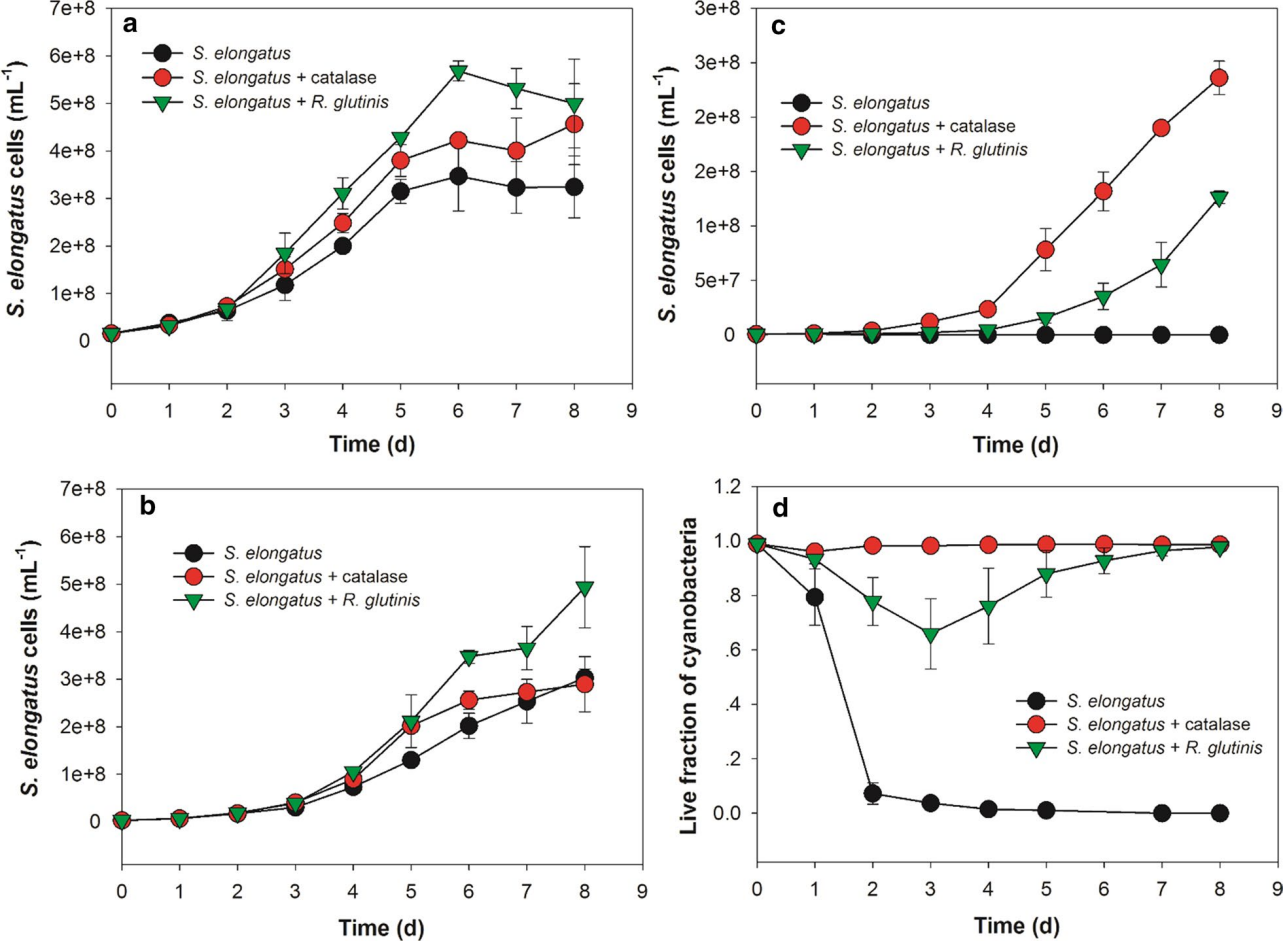
Growth of *S. elongatus* with/without yeast *R. glutinis*. **a** Initial cell number of *S. elongatus* was 1.6 × 10^7^/mL; **b** Initial cell number of *S. elongatus* was 3.2 × 10^6^/mL; **c** Initial cell number of *S. elongatus* was 1.6 × 10^6^/mL; **d** Live fraction of *S. elongatus cells* with initial cell number of 1.6 × 10^6^/mL. All data are averages of biological triplicates ± standard deviation

**Fig. 8:**
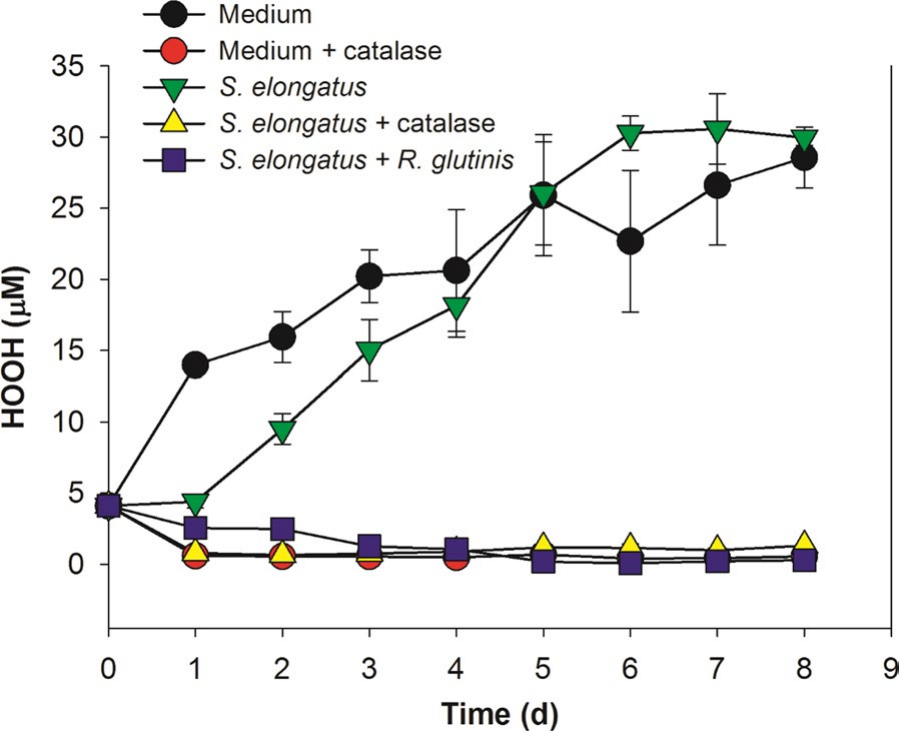
HOOH formation in HEPES buffered culture. Initial cell number of *S. elongatus* and *R. glutinis* was 1.6 × 10^6^ and 2 × 10^5^/mL, respectively. 1.25 mg/L of catalase was daily supplied. All data are averages of biological triplicates ± standard deviation

Hydrogen peroxide (HOOH) and related reactive oxygen species (ROS) can arise from a number of sources, including light exposure [38, 39]. Further, chemicals present in cultural systems are known to produce HOOH [40]. Previously, HEPES with 1-10 mM concentration in culture could generate enough HOOH to kill the axenic phytoplankter *Prochlorococcus* strain [40]. Indeed, in our cultures, BG-11[co] medium with various concentrations of HEPES produced increasing levels of HOOH (Fig. 6), with HOOH concentrations approaching an asymptotic level after several days. *S. elongatus* cells at various inoculum concentrations were tested to check the influence of HOOH on their axenic growth and to explore any role of yeast heterotrophs on the growth of the cyanobacterium under these conditions. At higher cell inoculum concentrations (1.6 × 10^7^ and 3.2 × 10^6^ cells/mL), the axenic growth of *S. elongatus* was robust (Fig. 7a, b), with negligible fractions of dead cells (data not shown). With a lower inoculum concentration of 1.6 × 10^6^ cells/mL, however, no growth of axenic *S. elongatus* culture was observed, and the fraction of live cells rapidly dropped to less than 10% after 2 days, and eventually all cells died (Fig. 7c, d). Correspondingly, the HOOH levels were kept below 1 µM in the cultures with higher cell inoculum concentrations (data not shown), but rapidly increased in the cultures with the lowest inoculum concentration, and reached 30 µM at the end of culture, showing a similar value as in BG-11[co] medium (Fig. 8). Although the unicellular cyanobacterium *S. elongatus* PCC 7942 possesses enzymes including ascorbate peroxidase and catalase to help to breakdown HOOH [41], the *S. elongatus* culture at the lowest cell inoculum was unable to prevent HOOH accumulation, likely resulting in the death of all *S. elongatus* cells.

Catalase was added in order to limit the impact of HOOH in pure cultures of *S. elongatus*. Contrary to BG-11[co] medium and in the pure cultures of *S. elongatus*, HOOH was kept at minimal concentrations following addition of catalase (Fig. 8). Importantly, *S. elongatus* grew better in the cultures with catalase, especially at an initial inoculum of 1.6 × 10^6^ cells/mL, while maintaining high viabilities (Fig. 7c, d). Also, catalase supplied to the axenic cultures of *S. elongatus* with higher inoculum concentrations of 1.6 × 10^7^ and 3.2 × 10^6^ cells/mL, exhibited a slightly enhancement in the growth and cell density during experimental growth.

Next, the impact of co-culturing with *R. glutinis* on *S. elongatus* growth and HOOH was investigated. The presence of the yeast *R. glutinis* partner clearly facilitated the growth of *S. elongatus* in co-cultures consistent with effects seen in dilute co-culture of cyanobacteria with other heterotrophs including *E. coli, B. subtilis,* and *S. cerevisiae* [22]. Cell numbers of *S. elongatus* with higher inoculum concentrations were significantly augmented in co-culture with *R. glutinis* compared with the axenic culture (Fig. 7a, b), even more than catalase alone. The growth enhancement was superior to the addition of catalase alone, suggesting that *R. glutinis* may be providing factors beyond HOOH reduction to the *S. elongatus* in co-culture. Even at the lowest inoculum concentration, the *S. elongatus* gradually grew in co-culture, albeit with a prolonged lag phase (Fig. 7c). Initially, the live fraction of *S. elongatus* cells decreased to approximately 60% by 3 days, but then gradually recovered to nearly 100% (Fig. 7d). At the same time, the HOOH content was progressively reduced and efficiently minimized to a similar low level as in BG-11[co] medium with catalase (Fig. 8).

The ability of pure cultures of *S. elongatus* to grow was strongly dependent upon the initial cell density, yet there was effectively no density dependence when catalase was supplied. These results along with the corresponding HOOH measurements indicated that HOOH accumulation in the illuminated cultures is likely to be a major cause of the toxicity effect during axenic growth of *S. elongatus*. However, *S. elongatus* in co-culture with *R. glutinis* could grow even at a dilute initial cell concentration, and the HOOH accumulation in the culture was efficiently reduced, suggesting that the eukaryotic heterotroph *R. glutinis* plays a key role in enabling its phototrophic partner’s growth by scavenging extracellular HOOH. Previous results have shown that dilute cultures of the marine cyanobacterium *Prochlorococcus* can be supported when co-cultured with certain marine heterotrophic bacteria [42, 43].

Furthermore, *R. glutinis* significantly boosts the growth of *S. elongatus* at high initial cell densities (Fig. 7), and the augmentation was higher than that obtained with addition of catalase. This increase indicates that the eukaryotic heterotroph may provide additional benefits to phototrophic partners in addition to HOOH elimination in order to support the growth of cyanobacteria even more. Previous studies have shown that the addition of thiosulfate, vitamin B12, biotin, and thiamine can be used to enhance and elevate the growth of the cyanobacterium *Synechococcus leopoliensis* CCAP1405/1 [44].

## Conclusions

Culture media formulations were obtained by evaluating the impact of medium components on axenic growth of the three yeast strains and the sucrose-secreting cyanobacterium *S. elongatus*. The yeasts *R. glutinis* and *C. curvatus* could efficiently take up sucrose secreted from *S. elongatus* and expand their cell numbers in co-culture, while *S. cerevisiae* cells failed to grow in co-culture with *S. elongatus* likely due to inefficient sucrose incorporation [21]. Furthermore, robust co-cultures of *R. glutinis* and *S. elongatus* could last for more than 1 month, with higher biomass and lipid yield than the axenic culture of *S. elongatus* in batch culture. These findings demonstrate the potential of a stable sustainable phototrophic-heterotrophic co-culture system as an emerging bioenergy platform, taking the advantage of synthesis of organic carbon from CO_2_ by photobionts and channeling the carbon to oleaginous heterotrophs without the need for exogenous supplies of carbon. Equally important, the phototroph and heterotroph both exhibited symbiotic interactions with their respective partner species. The cyanobacteria provide essential organic carbon nutrients to the yeast and perhaps other components, such as oxygen. Concomitantly, the yeast cells efficiently limit the generation of toxic ROS in the culture which enable and encourage survival and expansion of *S. elongatus* at low cell densities, significantly facilitating the growth of *S. elongatus* in co-cultures. Dealing with ROS may be a common feature defining community interaction between phototrophs and heterotrophs [45]. The finding that cyanobacterial growth in co-culture with yeast was superior to cultures containing catalase suggests additional factors gained from the heterotroph in co-culture. Two of the major constituents of some lichens include cyanobacteria and eukaryotic species [1, 2]. The results from the co-culture of cyanobacteria and fungal partner constructed in this study provide strong evidence that mutual interactions can be achieved between phototrophs and eukaryotic heterotrophs. While the phototroph provides an organic carbon source to heterotroph, the heterotroph can play an equally important role by assisting in phototroph survival and robust growth by protecting the cultures against oxidative stress. In this way, we have established a successful artificial lichen system composed of photobionts and eukaryotic heterotrophs yielding symbiotic and synergistic interactions that represent a potential sustainable production platform for biofuels from sunlight and CO_2_.

## Additional files

**Additional file 1.** Pure culture of cyanobacterium *S. elongatus* in BG-11 and BG-11 supplied with YE, respectively.

**Additional file 2.** The specific growth rate (per h) of different yeasts with varies concentrations of NaCl. Values shown are averages of biological triplicates ± standard deviation.

## Abbreviations

YNB w/o aa: yeast nitrogen base without amino acids
YE: yeast extract
ROS: reactive oxygen species
CO_2_: carbon dioxide
CFU: colony-forming units
HOOH: hydrogen peroxide
DW: dry weight
TFA: total fatty acids
