# Supplementary figures and images for: Mimicking lichens: incorporation of yeast strains together with sucrose-secreting cyanobacteria improves survival, growth, ROS removal, and lipid production in a stable mutualistic co-culture production platform
Source: Biotechnol Biofuels. 2017 Mar 21;10:55. doi: 10.1186/s13068-017-0736-x (PMC5360037; doi:10.1186/s13068-017-0736-x)

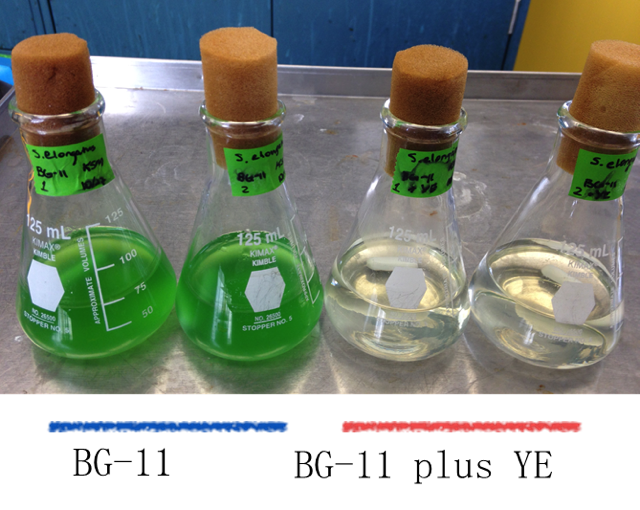

Supplement: Supplementary file 1 — Additional file 1. Pure culture of cyanobacterium S. elongatus in BG-11 and BG-11 supplied with YE, respectively. [file 13068_2017_736_MOESM1_ESM.docx]
