# Supplementary material for: Mimicking lichens: incorporation of yeast strains together with sucrose-secreting cyanobacteria improves survival, growth, ROS removal, and lipid production in a stable mutualistic co-culture production platform
Source: Biotechnol Biofuels. 2017 Mar 21;10:55. doi: 10.1186/s13068-017-0736-x (PMC5360037; doi:10.1186/s13068-017-0736-x)
Supplement: Supplementary file 2 — Additional file 2. The specific growth rate (per h) of different yeasts with varies concentrations of NaCl. Values shown are averages of biological triplicates ± standard deviation. [file 13068_2017_736_MOESM2_ESM.docx]

| **NaCl (mM)** | **Specific growth rate (per h) of yeasts** | | |
| --- | --- | --- | --- |
|  | ***C. curvatus*** | ***R. glutinis*** | ***S. cerevisiae*** |
| 0 | 0.30 ± 0.01 | 0.20 ± 0.01 | 0.15 ± 0.00 |
| 100 | 0.29 ± 0.00 | 0.17 ± 0.01 | 0.07 ± 0.00 |
| 150 | 0.25 ± 0.01 | 0.14 ± 0.00 | 0.07 ± 0.00 |
| 200 | 0.19 ± 0.00 | 0.13 ± 0.00 | 0.05 ± 0.00 |
